# Genes uniquely expressed in human growth plate chondrocytes uncover a distinct regulatory network

**DOI:** 10.1186/s12864-017-4378-y

**Published:** 2017-12-20

**Authors:** Bing Li, Karthika Balasubramanian, Deborah Krakow, Daniel H. Cohn

**Affiliations:** 1 Department of Molecular, Cell, and Developmental Biology, University of California Los Angeles, CA, Los Angeles USA; 2 Department of Orthopaedic Surgery, David Geffen School of Medicine at the University of California Los Angeles, CA, Los Angeles USA; 3 Department of Obstetrics and Gynecology, David Geffen School of Medicine at the University of California at Los Angeles, CA, Los Angeles USA; 4 Department of Human Genetics, David Geffen School of Medicine at the University of California Los Angeles, CA, Los Angeles USA

**Keywords:** Cartilage, Chondrocyte, RNA-seq, lncRNA, Skeletal dysplasia, Gene expression, Human

## Abstract

**Background:**

Chondrogenesis is the earliest stage of skeletal development and is a highly dynamic process, integrating the activities and functions of transcription factors, cell signaling molecules and extracellular matrix proteins. The molecular mechanisms underlying chondrogenesis have been extensively studied and multiple key regulators of this process have been identified. However, a genome-wide overview of the gene regulatory network in chondrogenesis has not been achieved.

**Results:**

In this study, employing RNA sequencing, we identified 332 protein coding genes and 34 long non-coding RNA (lncRNA) genes that are highly selectively expressed in human fetal growth plate chondrocytes. Among the protein coding genes, 32 genes were associated with 62 distinct human skeletal disorders and 153 genes were associated with skeletal defects in knockout mice, confirming their essential roles in skeletal formation. These gene products formed a comprehensive physical interaction network and participated in multiple cellular processes regulating skeletal development. The data also revealed 34 transcription factors and 11,334 distal enhancers that were uniquely active in chondrocytes, functioning as transcriptional regulators for the cartilage-selective genes.

**Conclusions:**

Our findings revealed a complex gene regulatory network controlling skeletal development whereby transcription factors, enhancers and lncRNAs participate in chondrogenesis by transcriptional regulation of key genes. Additionally, the cartilage-selective genes represent candidate genes for unsolved human skeletal disorders.

**Electronic supplementary material:**

The online version of this article (10.1186/s12864-017-4378-y) contains supplementary material, which is available to authorized users.

## Background

Chondrogenesis is the earliest stage of skeletal development and begins during embryogenesis with the proliferation and condensation of mesenchymal cells, followed by lineage commitment and differentiation, to yield the chondrocytes which populate and synthesize the scaffold for patterning the early skeletal elements. Subsequently, growth and mineralization of endochondral bones proceeds by establishment of the growth plates followed by the ordered differentiation, proliferation and hypertrophy of growth plate chondrocytes. Most of the terminally differentiated hypertrophic chondrocytes undergo apoptosis, while a subset can transdifferentiate to osteoblasts [1, 2]. Finally, the cartilage matrix is mineralized to form bone. This highly dynamic developmental process is regulated by transcription factors that include SOX9, RUNX2 and the GLI family of proteins, with synergistic regulation by multiple cell signaling pathways including the fibroblast growth factor (FGF), hedgehog, bone morphogenetic protein (BMP), transforming growth factor beta (TGF-beta) and WNT signaling pathways [3, 4]. Disruption of the genes that participate in chondrogenesis can lead to inherited skeletal disorders, as observed in humans and mice, which are characterized by abnormalities in the formation, linear growth and/or maintenance of the skeleton. The skeletal disorders comprise a broad range of severity from embryonic lethal phenotypes with extensive skeletal abnormalities to mild disorders in which short stature and early onset osteoarthritis are the main manifestations [5, 6].

Systematic identification of genes expressed in cartilage can uncover gene regulatory networks that are important for chondrogenesis and chondrocyte biology, providing fundamental insight into cartilage development and function. Indeed high-throughput gene expression profiling followed by data mining has been used to identify tissue-specific/selective genes in multiple human tissues, including cartilage [7–11]. Studies in cartilage have discovered genes that are highly and/or primarily expressed in chondrocytes and have been used as markers to investigate the differentiation potential of mesenchymal stem cells toward chondrocytes and other cell lineages [12]. Not surprisingly, a large number of the genes selectively expressed in chondrocytes are associated with human skeletal disorders [7], and the published gene expression data have been used as a resource to facilitate studies that have led to identification of additional genes associated with human skeletal phenotypes. For example, *TRPV4*, *COL11A1* and *ACAN* gene mutations were identified as the causes of autosomal dominant brachyolmia, fibrochondrogenesis and an autosomal recessive form of spondyloepimetaphyseal dysplasia, respectively, based in part on their cartilage-selective (CS) expression patterns [13–15].

Microarray technology has been widely used for the high-throughput detection of gene expression. However, microarray gene expression applications are limited by low signal-to-noise ratios for genes expressed at low levels, signal saturation for highly expressed genes, and detection restricted to a predefined gene set. The development of high throughput sequencing methods has facilitated measuring most cellular transcript levels by RNA sequencing (RNA-Seq) [16]. In contrast to microarray technology, RNA-seq provides more sensitive and accurate measurement of gene expression, and is able to discover novel transcripts and genes. To provide a deeper measure of both quantitative and qualitative gene expression in human growth plate chondrocytes, we used RNA-seq to acquire a comprehensive gene expression profile of the tissue. These data were complemented by publically available RNA-Seq and ChIP-seq data sets, also generated on high throughput sequencing platforms, to reveal regulatory mechanisms that underlie chondrogenesis and linear growth. The data have provided a comprehensive understanding of the transcriptional network in chondrocytes and suggested novel regulatory networks and mechanisms underlying chondrogenesis and skeletal development.

## Results

### Identification of cartilage-selective (CS) genes in human fetal cartilage

We sought to identify CS genes using RNA-seq data from human tissues and cells. Cartilage gene expression profiles were generated from 14 to 18 week distal femur growth plate chondrocytes. RNA-seq data from other tissues/cells were generated by previously described studies [17–23] and accessed from public databases as detailed in Methods. There were 111 RNA-seq gene expression datasets representing 22 tissues or cell types in the comparison data set (Additional file 1: Dataset S1). With the exception of the data from CD4+ and CD8+ primary cells, which were obtained from adults, the remaining data were generated from human fetal tissues/cells. Therefore, the gene expression data mainly reflected human fetal transcription. Data quality were assessed by calculating the Pearson’s correlation coefficients between datasets, requiring that gene expression patterns be similar in the same or closely related tissues, which would be reflected by high Pearson’s correlation coefficients. As shown in Additional file 2: Figure S1, tissues/cells with same origins had high Pearson’s correlation coefficients and formed unique clusters, indicating that the data were of high quality and therefore represented the intrinsic gene expression properties of the tissues/cells studied.

Using the PaGeFinder algorithm, we identified 403 transcripts, representing 332 protein coding genes, which were selectively expressed in cartilage (Additional file 3: Dataset S2). A boxplot of the selective gene expression showed that there was a set of genes that were highly expressed in cartilage as compared with other tissues/cells (Additional file 2: Figure S2). Many well-studied cartilage marker genes were identified and listed as top CS genes with high specificity measure (SPM) values, such as the known CS genes encoding types II, IX and XI collagens, aggrecan and matrix metalloproteinase 13 (Additional file 3: Dataset S2). We found that 63 (42%) of the CS genes previously discovered in our similar microarray-based study [7] were also identified as CS in the current study (Additional file 4: Dataset S3). Another 25 genes from the previous study [7] had high SPM values (between 0.7 and 0.9) in the current study, indicating they were preferentially, but not selectively, expressed in cartilage. This comparison indicates that the RNA-seq approach was capable of recapitulating the prior findings [7] and able to reveal known and newly identified genes selectively expressed in human fetal cartilage. There were also 57 CS genes from the microarray study that showed expression in non-cartilage samples in the RNA-seq dataset, reflecting the differences between the microarray and RNA-seq techniques. RNA-seq has a broader dynamic range that is able to better detect low-abundance transcripts [24]. Thus genes expressed at low levels on microarrays have signals close to background and are set as non-expressed while by RNA-seq the low level expression can be measured, revealing that they may not be selectively expressed.

**Fig. 1:**
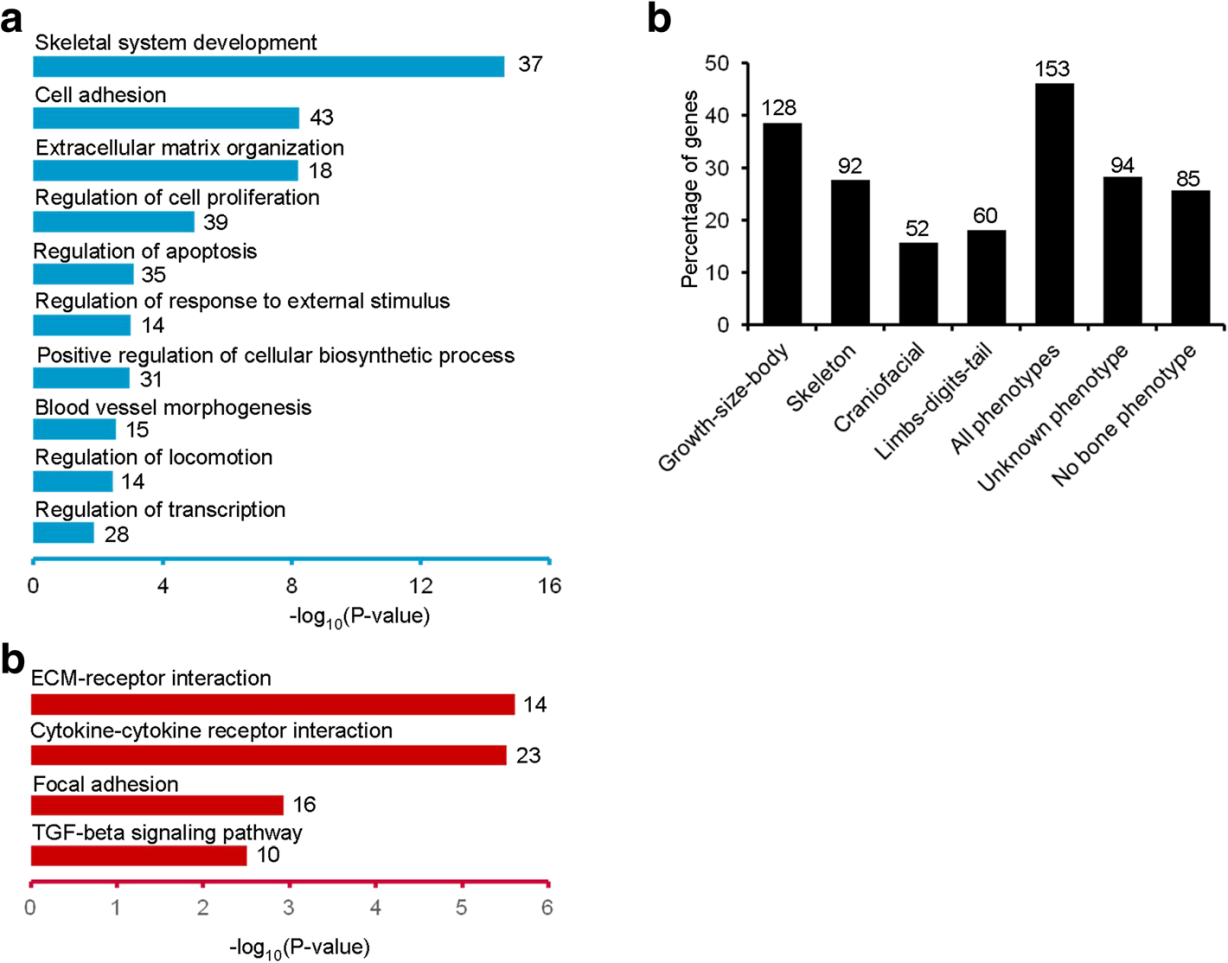
Cartilage-selective genes are required for proper bone formation. **a** Gene ontology (GO) analysis of cartilage-selective (CS) genes. **b** KEGG pathway analysis of cartilage-selective genes. The number of genes in each GO term is indicated. **c** CS genes associated with skeletal phenotypes in gene targeted mice

GO and KEGG pathway analysis indicated that the cartilage-selective gene set was enriched for genes known to participate in skeletal system development. These included selectively expressed genes involved in cellular processes necessary for chondrogenesis, such as regulation of cell proliferation and apoptosis, cellular biosynthesis, cell adhesion, blood vessel morphogenesis, transcriptional regulation and TGFβ signaling (Fig. 1a, b).

### Cartilage-selective genes are required for skeletal development

As the GO analysis of the CS genes indicated that they have roles in skeletal development, it would be expected that disruption of their functions in mice and/or humans would lead to skeletal abnormalities. To assess this, we retrieved mouse phenotypes from the Mouse Genome Informatics Database (MGI, http://www.informatics.jax.org/) and found that mutations in 153/332 (46%) of the CS genes were associated with mouse skeletal defects (Fig. 1c; Additional file 3: Dataset S2). Using the MGI descriptors, the 153 genes were represented across a number of overlapping categories, with 128 genes associated with growth/size/body phenotypes, 92 genes associated with skeletal phenotypes, 52 genes associated with craniofacial phenotypes, and 60 genes associated with limb/digit/tail phenotypes. For the remaining 179 genes, 94 genes were not present in the MGI phenotype list and 85 genes did not show a skeletal phenotype when targeted in mice. Despite the latter observation, a review of the literature revealed that many of these genes do have roles in skeletogenesis, including human skeletal disorders due to mutations in *SHOX*, *FBLN7* and *VIT* as examples [25–27]. In addition, although knockout mutations targeting *Trpv4* or *Comp* do not show skeletal phenotypes in mice, dominant negative structural mutations in these genes result in skeletal disorders in humans [14, 28–30]. In this context, we compared the CS genes with the genes associated with human genetic skeletal disorders, expecting that many of the genes would be associated with skeletal dysplasias [5]. There were 32 of the CS genes (Additional file 3: Dataset S2) associated with a broad spectrum of 62 human skeletal disorders, 26 of which were also associated with mouse skeletal phenotypes. The human and mouse skeletal phenotypes highlight the essential roles of the CS genes in skeletal development but also reveal that loss-of-function mouse models do not identify the essential roles of all genes important for skeletogenesis.

### Cartilage-selective gene products interact

**Fig. 2:**
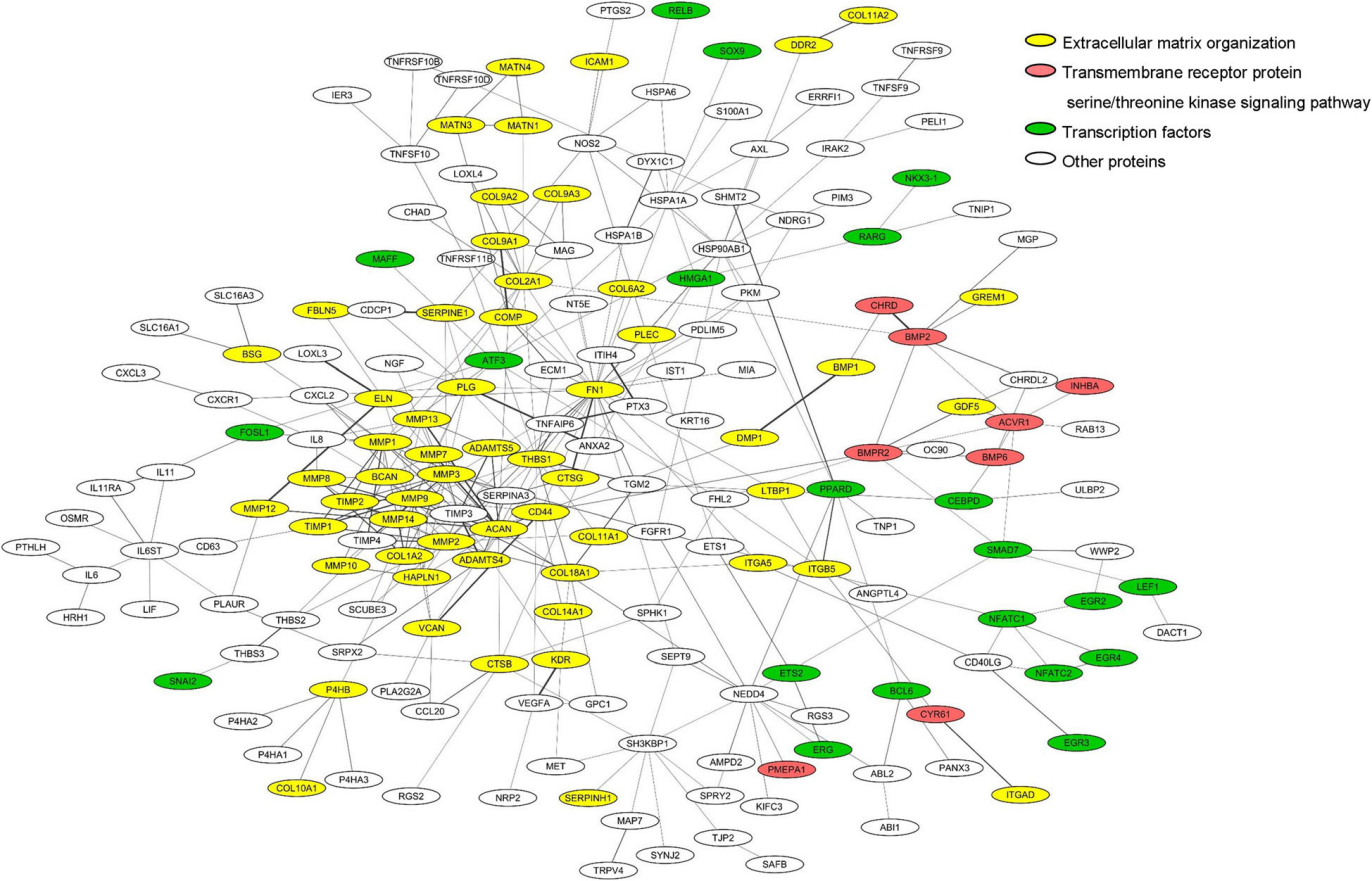
Protein-protein interaction network for the CS genes. CS proteins are represented as colored nodes. Node colors represent three GO groups as noted

To decipher relationships among the proteins encoded by the selective genes, a protein-protein interaction network was built with Cytoscape [31]. As shown in Fig. 2, proteins encoded by the genes showed extensive physical interactions and formed a large interaction network. Matrix metallopeptidases had complex interactions with extracellular matrix proteins, such as CS collagens and aggrecan, consistent with their central roles in processing these proteins and regulating bone mineralization. TGFβ family signaling molecules including BMP2/6, ACVR1 and GDF5 also showed physical interactions (Fig. 2), in agreement with their regulatory functions in chondrogenesis and bone formation [32]. Thus the proteins specified by the CS genes comprise a functional cartilage interactome.

### Transcriptional regulation of cartilage-selective genes

**Fig. 3:**
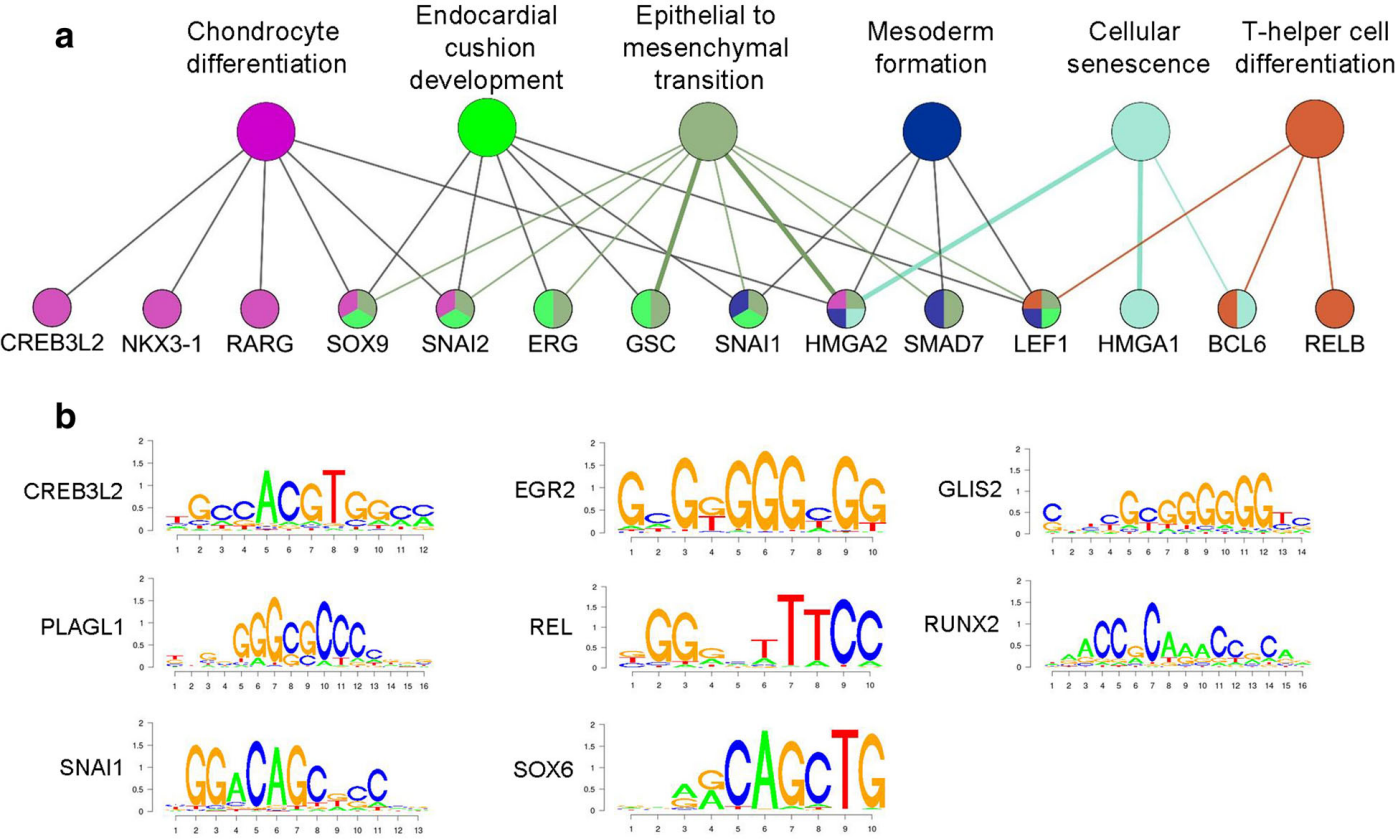
Cartilage-selective transcription factors and DNA binding motif analysis. **a** ClueGO network analysis for enrichment biological process modules within transcription factors. GO terms are represented as large nodes and genes are represented as small nodes. Node colors represent GO groups. **b** Transcription factor DNA binding motif analysis shows enrichment of motifs in cartilage-selective gene promoters. All *P* values for motif enrichment are less than 10^−10^

We expected that CS transcription factors could function as master regulatory molecules to control downstream expression of additional selectively expressed genes. By comparing the CS genes with the transcription factor genes annotated in the Animal Transcription Factor Database (AnimalTFDB) [33], 34 selectively expressed cartilage transcription factors were identified [Figs. 1a, 2; Additional file 5: Dataset S4]. These transcription factors are known or now predicted to participate in multiple cellular activities that regulate chondrocyte development (Fig. 3a). To determine if these factors control CS gene transcription, we searched for transcription factor binding motifs in the promoter regions of the CS genes. As shown in Fig. 3b, the promoters were enriched in consensus-binding sites for transcription factors that were selectively expressed in cartilage, including SOX6, CREB3L2, EGR2/3/4, GLIS3, PLAGL1, RELB and SNAI1/2, all of which have been shown to play important roles in skeletal development [34–39]. Although *RUNX2* was not identified as a CS gene, since it was also expressed in lymphocytes (Additional file 2: Figure S3), its binding motif was also enriched among the CS promoters (Fig. 3b), consistent with its known transcriptional regulatory role in chondrogenesis. These data suggest that expression of the selective genes might be directly regulated by a group of transcription factors that are uniquely expressed in cartilage.

**Fig. 4:**
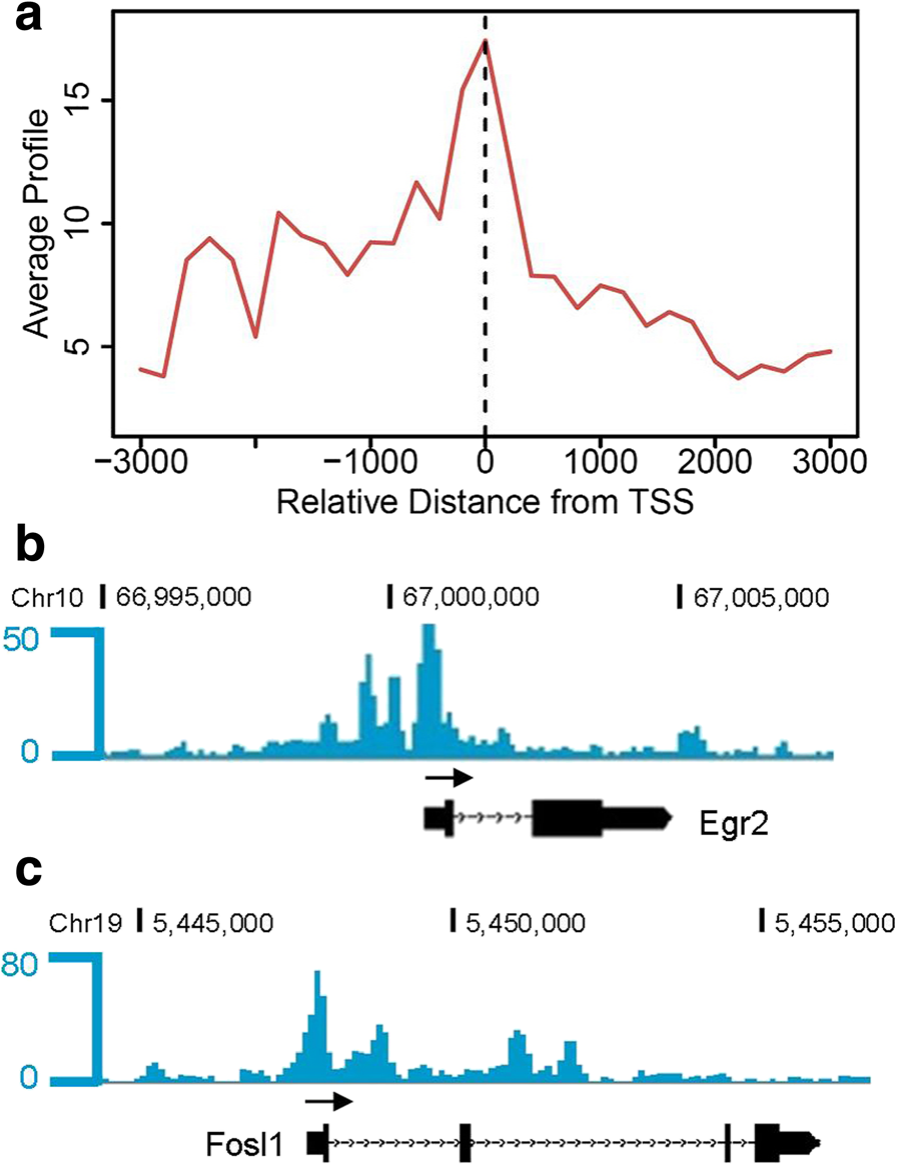
Sox9 binds to the cartilage-selective transcription factor gene promoters in mouse chondrocytes. **a** Average binding profile of Sox9 at the CS transcription factor gene TSS. The distance on the plot is measured in base pairs. **b, c** Genome browser views of Sox9 peaks along the *Egr2* **b** and *Fosl1* **c** loci

The binding motif of SOX6 was also enriched among the CS gene promoters, and SOX6 cooperates with SOX5 to secure SOX9 binding to CS enhancers in chondrocytes [40]. *SOX9* is expressed in skeletogenic mesenchymal cells at early embryonic stages and regulates chondrogenesis through transcriptional activation of target genes [41, 42]. *SOX9* mutations in humans produce campomelic dysplasia, a generally lethal osteochondrodysplasia with distinctive abnormalities in cartilage and bone [43, 44]. As has been previously observed in animal models, *SOX9* is selectively expressed in cartilage and is required for expression of CS genes in chondrocytes [41, 42, 45]. In our analysis, 247 (74.4%) CS genes had SOX9 binding sites in their promoter regions (±2 kb of TSS). These CS genes included 25 transcription factor genes which by ChIP-seq had SOX9 bound to their TSS (Fig. 4a), with 13 of them showing strong SOX9 binding intensity (>25% quantile of all SOX9 binding sites). For instance, SOX9 strongly binds to the TSS of transcription factor genes *Egr2* and *Fosl1* (Fig. 4b, c), which are essential for skeletogenesis in mice [36, 46]. Because both *SOX9* and *EGR2* are highly expressed and regulate gene expression in mesenchymal stem cells (MSC) [41, 42, 47, 48], it is plausible to speculate that SOX9 controls chondrogenesis through functions as an upstream transcription factor that directly activates expression of downstream transcription factors.

### Cartilage-selective genes are regulated by active enhancers

**Fig. 5:**
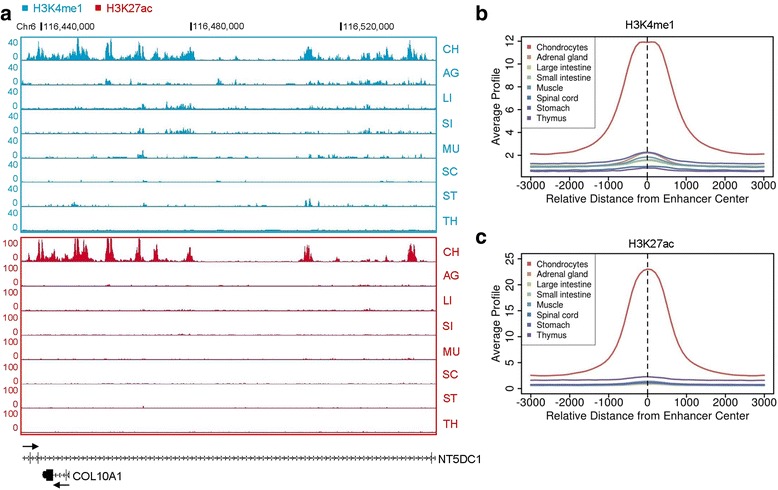
Identification of active enhancers in chondrocytes. **a** Genome browser views of H3K4me1 and H3K27ac peaks along the *COL10A1* locus on chromosome 6. Chromosome coordinates are shown as black bars on top. Black arrows indicate the direction of transcription on a diagram of the gene below. CH, chondrocytes. AG, adrenal gland. LI, large intestine. SI, small intestine. MU, muscle. SC, spinal cord. ST, stomach. TH, thymus. **b** and **c** Genome-wide average of **b** H3K4me1 and **c** H3K27ac enrichment at chondrocyte enhancers in human fetal tissues and cells. The distance on the plot is measured in base pairs

**Fig. 6:**
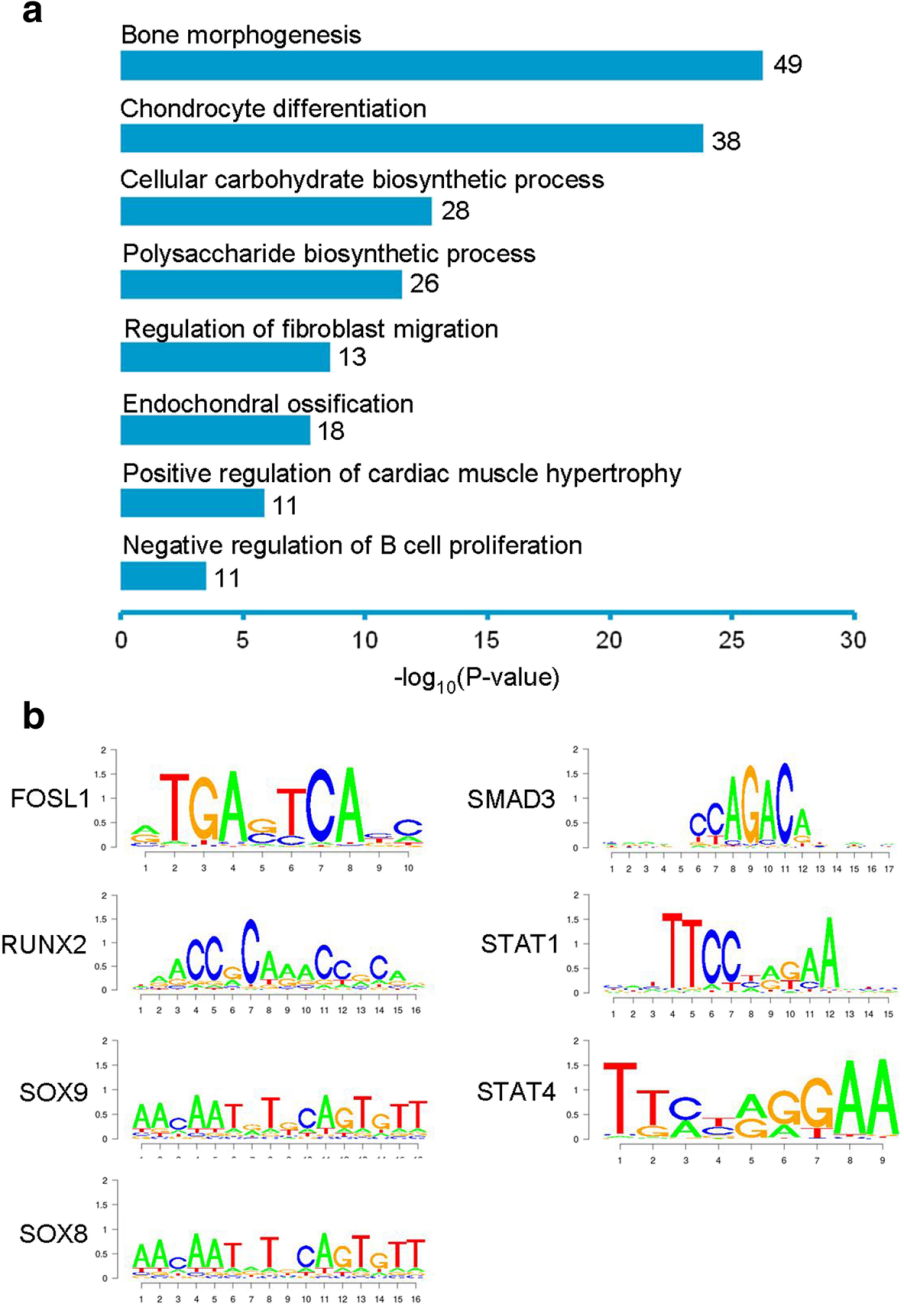
GO and DNA binding motif analysis of chondrocyte-unique enhancers. **a** GO analysis of chondrocyte enhancers. The number of genes in each GO term is indicated. **b** Enrichment of transcription factor DNA binding motifs on chondrocyte enhancers. All *P* values for motif enrichment are less than 10^−10^

Spatiotemporal gene expression is modulated by both transcription factors and epigenetic mechanisms such as enhancers [49]. Enhancers are distinct genomic regions bound by transcriptional activators and are marked by histone modifications such as histone H3 lysine 4 monomethylation (H3K4me1) and lysine 27 acetylation (H3K27ac) [50, 51]. Activation of tissue-specific enhancers provides a molecular mechanism that turns on gene expression in a cell type-specific manner [49]. Genome-wide studies in chondrocytes have identified a set of genes involved in chondrogenesis that are regulated by enhancers [52–54], so we asked whether CS gene expression might be activated by enhancers. Active enhancers in human chondrocytes were predicted by mapping the genome-wide distribution of H3K27ac [55], revealing 31,235 active chondrocyte enhancer regions marked by this chromatin modification (Additional file 6: Dataset S5). By comparing these data with enhancers across seven fetal tissues, we identified a subset of 11,334 enhancers that were uniquely present in chondrocytes. These enhancers were specifically enriched at CS gene loci including *COL10A1*, *COL2A1* and *COL11A1* (Fig. 5a and Additional file 2: Figure S4). Similar to other tissues, the chondrocyte enhancers were predominantly bound by H3K4me1 and H3K27ac (Fig. 5b, c). To predict enhancer target genes, we examined genome-wide enhancer-gene associations in chondrocytes with GREAT [56]. As shown in Fig. 6a, the chondrocyte unique enhancers were associated with genes involved in skeletal developmental processes including bone morphogenesis, chondrocyte differentiation, cellular carbohydrate biosynthesis, polysaccharide biosynthesis and endochondral ossification. 176 (53%) of the CS genes had active chondrocyte unique enhancers flanking their loci, suggesting that these genes are potentially enhancer regulated.

With DNA motif analysis, we found that the chondrocyte unique enhancers were enriched for consensus-binding sites for transcription factors that have pivotal roles in chondrogenesis, including RUNX2, SOX8/9, SMAD3, STAT1/4 and FOSL1 (Fig. 6b), in agreement with the fact that they regulate transcription in part through binding to enhancers [52, 54, 57–60]. Furthermore, binding sites for additional transcription factors were also found to be enriched among the chondrocyte unique enhancers, suggesting roles in transcriptional regulation in chondrocytes that could be further explored experimentally (Additional file 6: Dataset S5).

### Identification of cartilage-selective lncRNAs

**Fig. 7:**
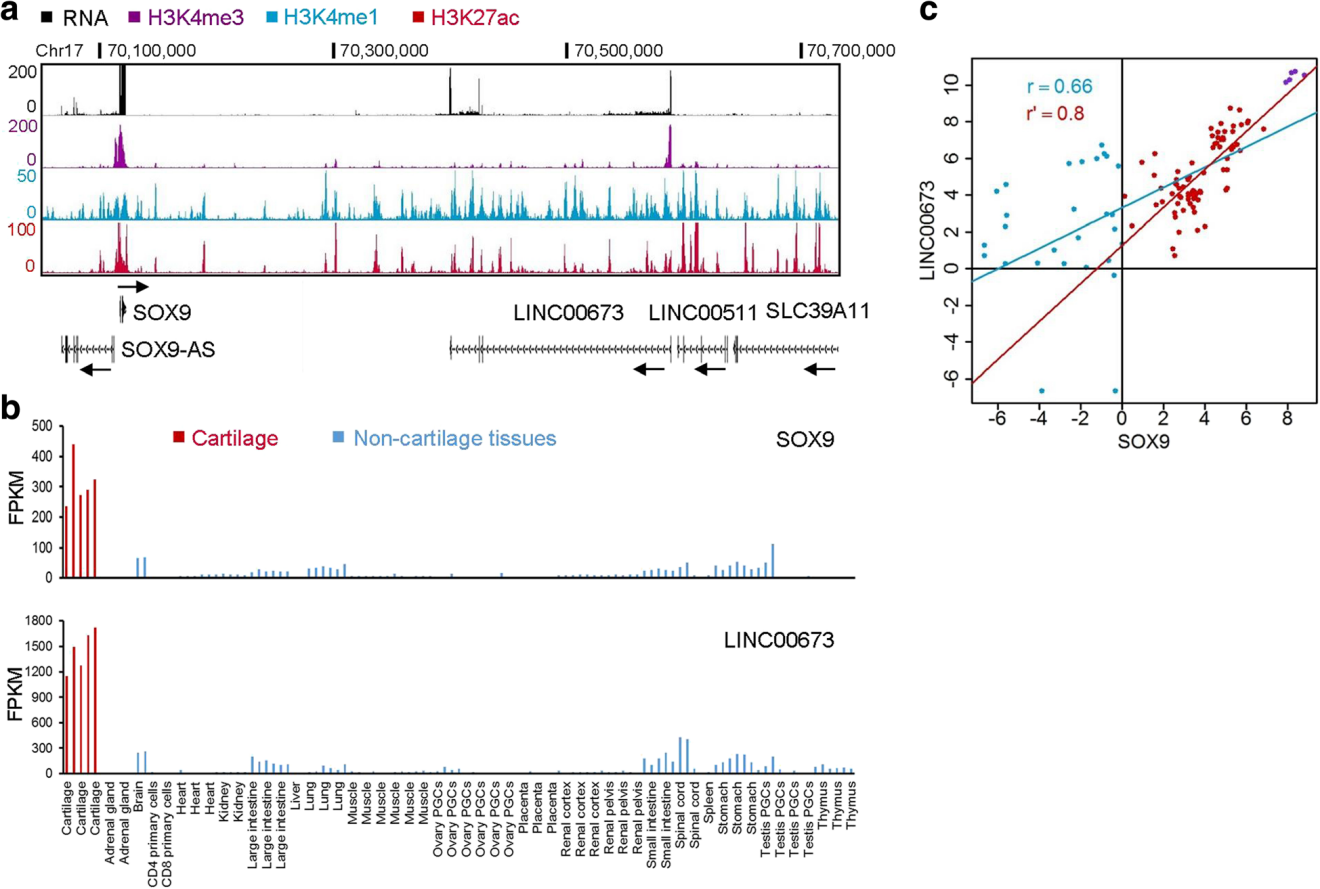
*LINC00673* is co-expressed with *SOX9*. **a** Genome browser views of H3K4me1, H3K4me3, H3K27ac peaks and RNA expression track along *SOX9* and *LINC00673* loci on chromosome 17 in human chondrocytes. **b** Bar graphs showing *SOX9* and *LINC00673* expression in human tissues. Red bars indicate cartilage samples and blue bars indicate non-cartilage samples, respectively. Gene expression levels are shown as FPKM values. **c** Correlation between *SOX9* and *LINC00673* expression (Log_2_FPKM) in human tissues. Scatter plot of *SOX9* against *LINC00673* with regression lines showing a correlation between the two genes using the Pearson’s correlation coefficient. Red and blue dots indicates the tissues with *SOX9* Log_2_FPKM value greater or less than 1, respectively. Cartilage samples are highlighted as purple dots. The blue regression line was generated with data from all tissues. The red regression line was generated with data from the tissues that have *SOX9* FPKM value ≥1. The Pearson’s correlation coefficient for each regression line is shown

LncRNAs are emerging as transcriptional regulators that participate in skeletogenesis through regulation of chondrocyte and osteoblast differentiation and homeostasis [61]. Since most lncRNAs are polyadenylated, and therefore can be measured by mRNA-seq, we analyzed the RNA-seq data and identified 34 lncRNAs genes selectively expressed in cartilage. LncRNAs have been shown to act as cis-regulators that modulate expression of neighboring genes [62, 63]. To identify potential regulatory targets, we used GREAT analysis to reveal protein coding genes neighboring the cartilage selective lncRNAs (Additional file 7: Dataset S6). Eight lncRNAs were adjacent to genes known to be involved in skeletal development including *SOX9*, *GLI2*, *FOXA2*, *UMPS*, *SOD2*, *BHLHE41* and *RAB11FIP4*. Three lncRNAs (*LINC00673, AC058791.1* and *RP11–261P24.2*) were embedded in active enhancer regions that were marked by H3K27ac and H3K4me1 in chondrocytes (Fig. 7a; Additional file 2: Figure S5), suggesting the lncRNA expression and enhancer activity might be functionally related. Among these, we were particularly interested in *LINC00673* (annotated as a fusion gene with LINC00511 by ENSEMBL) because it is transcribed from a locus on human chromosome 17 that is ~280 kb downstream of *SOX9*. In human chondrocytes, *LINC00673* was highly expressed and its promoter was enriched in H3K4me3, a marker for active transcription. The active enhancers at the *LINC00673* gene locus span a ~0.5 Mb region downstream of *SOX9*. This region and the upstream region of *SOX9* were marked with H3K27ac and shown to interact with the *SOX9* promoter in melanoma cells that express SOX9 [64]. These data suggest that the *LINC00673* locus might function as an enhancer to regulate *SOX9* transcription (Fig. 7a).

*LINC00673* and *SOX9* were highly expressed in chondrocytes, and also had low expression in brain, kidney, intestine, lung, spinal cord and stomach (Fig. 7b). Among all tissues, the expression levels of *LINC00673* and *SOX9* were positively correlated with each other, yielding a Pearson’s correlation coefficient 0.66. When we only considered the tissues/cells that express *SOX9* (FPKM > 1), the Pearson’s correlation coefficient reached 0.8, indicating *LINC00673* and *SOX9* have orchestrated expression patterns across tissues (Fig. 7c). To validate the finding, we analyzed gene expression data using the UCLA Gene Expression Tool (UGET) [65], which reported the Pearson’s correlation coefficient for every pair of 54,675 probe sets on Affymetrix HG U133 Plus 2.0 arrays from the ∼12,000 human expression profiles. Among the 38,500 genes detected by the Affymetrix arrays, *SOX9* was ranked as the gene with the second highest correlation with *LINC00673* expression (the most highly correlated gene was *LINC00673* itself), with a Pearson’s correlation coefficient 0.43. Reciprocally, *LINC00673* was ranked as the gene that had the nineteenth highest correlation with SOX9 expression, with a Pearson’s correlation coefficient 0.39. The high Pearson’s correlation coefficients suggest a gene expression co-regulatory mechanism between *SOX9* and *LINC00673* (Additional file 7: Dataset S6)*.*

Because SOX9 positively regulates transcription through binding to gene promoters and enhancers [54], we also considered the possibility that SOX9 could directly bind to *LINC00673* and activate its transcription. To assess this, we examined SOX9 binding sites in mouse chondrocytes [52] and found that SOX9 bound to the promoter and structural gene for *2610035D17Rik,* the orthologue of *LINC00673* in mice, strongly suggesting that expression of the gene is regulated by SOX9 (Additional file 2: Figure S6). Because *LINC00673* was selectively expressed in chondrocytes and was embedded in the enhancer region downstream of *SOX9* gene, it is also possible that there is reciprocal regulation in which *LINC00673* could work with enhancers to activate *SOX9* and possibly other genes, and therefore participate in regulation of chondrogenesis.

## Discussion

Organogenesis is a process of proliferation and differentiation of progenitor cells into diverse types of cells with distinct functions. Their unique functions are largely acquired through expression of tissue-specific/selective genes. Studies of gene expression patterns and regulatory mechanisms in particular tissues thus inform our understanding of tissue development and mechanisms of disease. Tissue-specificity refers to genes whose expression is restricted to one particular tissue or cell type, while tissue-selective expression includes genes that are expressed in one or a few generally biologically similar tissue types [66]. The data presented in this study described 332 cartilage-selective genes expressed in human fetal cartilage, providing unique insights into chondrocyte gene expression and regulation.

### The significance of cartilage-selective gene identification

High expression of the CS genes in chondrocytes suggests they may have essential roles in skeletal development and skeletal disorders. In support of this hypothesis, 32 CS genes were linked to human skeletal disorders that result from mutations in these genes. Gene-targeting mice for 153 genes showed skeletal development defects. Among the list, there were well-known genes whose products have pivotal roles in chondrogenesis, such as cartilage collagens, cell signaling molecules and transcription factors. Base on this observation, we suggest that some of the newly identified CS genes may be good candidate genes for skeletal disorders in which the associated genes have yet to be identified. As new skeletal dysplasia loci are defined, the candidate genes can be compared with the CS genes to promote rapid identification of the mutated gene. Gene network analysis revealed complex interactions of CS gene products, data compatible with the hypothesis that CS genes are functionally related in the initiation and progression of complex diseases such as osteoarthritis. It may thus be useful to correlate gene mutations and dysregulation of gene expression among the chondrodysplasias with the CS gene interactome for further understanding of the pathogenesis of cartilage disorders [67]. In addition, we identified 94 genes that are less studied and for which knockout mice phenotypes have not been annotated by MGI (Additional file 3: Dataset S2). According to GO analysis, 39 of these genes are involved in cell signaling pathways and 27 genes encode secreted proteins. Their unique cartilage expression pattern suggests the genes might participate in chondrogenesis. For example, *C2orf82* is highly expressed in human cartilage with an average FPKM value >1500 and encodes a type I single-pass transmembrane chondroitin sulfate proteoglycan. The gene showed a CS expression pattern with the highest expression in proliferating and prehypertrophic zones in embryonic and postnatal growth plate. Its expression was restricted to the uncalcified zone in adult articular cartilage, including chondrocyte clusters in human osteoarthritic cartilage. Its expression pattern was similar to the highly expressed CS genes *Sox9*, *Acan* and *Col2a1*, and could be induced by BMP-2, suggesting its functions may be associated with the development and maintenance of the chondrocyte phenotype [68]. As a second example, zinc-finger 385 family genes including ZNF385B/C/D were also selectively expressed in cartilage and another family member, ZNF385A, was also highly expressed in the tissue (Additional file 3: Dataset S2). Fluorescence-labeled Znf385C protein was highly expressed in maturing chondrocytes in the pharyngeal arches as well as in the cartilage associated with the ear and ventral fins in zebrafish [69]. Loss of *Znf385C* in zebrafish led to craniofacial defects and premature ossification of the Meckel’s cartilage, potentially through disturbance of p53-dependent cell cycle regulation [69]. Therefore, targeting some of the 94 understudied CS genes is likely to reveal new biology related to cartilage function.

### Regulation of chondrogenesis by epigenetic mechanisms

Epigenetic mechanisms such as histone modifications and enhancers regulate transcription in skeletal development and disease [70, 71]. Genome-wide profiling of SOX9 indicated that it functions in part by binding to enhancers. Disruption of upstream distal intergenic regions especially the regions between -50 kb and -350 kb 5′ of *SOX9* by deletion/translocation/inversion are associated with severe campomelic dysplasia [72, 73]. Systematically screening the 350 kb region upstream of *SOX9* using histone modification markers and DNase I hypersensitivity assays identified ten potential enhancers, with three enhancers primarily active in chondrocytes and that activate *SOX9* expression in the chondrocyte lineage [74]. SOX9, in complex with SOX5 and SOX6, reciprocally activate the enhancers, which provides a potential mechanism for autoregulation of its own expression in chondrocytes [74]. In the current study, genome-wide mapping of histone marks led to identification of 11,334 enhancers that were uniquely active in chondrocytes and associated with 176 of the CS genes. The identified active enhancers could be used as a resource to explore epigenetic regulation and higher-order chromatin organization in chondrocytes. The data also indicated that these enhancers were enriched in the consensus binding motifs for well-known transcription factors such as SOX9 and RUNX2, suggesting that transcription factor-bound enhancers contribute to the selective gene expression pattern in chondrocytes.

### lncRNAs and chondrogenesis

lncRNAs are non-protein coding transcripts with lengths of more than 200 nucleotides, which are proposed to regulate transcription through both *trans*- and *cis*-regulatory mechanisms. Some lncRNAs tether to their target gene loci and affect transcriptional activity of neighboring genes through association with regulatory proteins. They could also bind to regions on other chromosomes and also control gene activity through recruiting regulatory proteins [75]. The roles for lncRNAs in skeletal development are emerging with a growing number of lncRNAs identified that participate in skeletal development and disease [61]. In our study, 34 lncRNAs were identified to be selectively expressed in human cartilage. Eight lncRNAs are adjacent to loci of protein coding genes that participate in skeletal development, suggesting the cartilage-selective lncRNAs may be involved in chondrogenesis through regulation of protein coding genes. We found that *LINC00673* and *SOX9* are co-regulated in human tissues, and that *LINC00673* is embedded within a potential enhancer region for *SOX9*, suggesting LINC00673 may regulate *SOX9* expression through a distal enhancer. In support of this hypothesis, by Circularized Chromosome Conformation Capture sequencing (4C–seq), the *LINC00673* locus was found to be involved in long-range enhancer-promoter interactions at the *SOX9* locus in *SOX9*-expressed melanoma cells but not in *SOX9*-inactive cells, consistent with the enhancer-promoter interactions driving *SOX9* activation [64]. In addition, *LINC00673* regulates target gene expression in gastric cancer cells by recruiting histone methyltransferase EZH2 and demethylase LSD1 to target gene promoters, suggesting that it can function as a transcriptional regulator through epigenetic mechanisms [76]. While the exact role of *LINC00673* in *SOX9* gene regulation is unknown, loss- and gain-of-function experiments could be used to directly address this question.

### Cartilage-selective genes and mouse phenotypes

There were 85 CS genes that did not show a skeletal phenotype when targeted in mice (Figure 1C). Some of these genes may have overlapping functions with paralogous genes. For example, NKX3.1 is a transcription factor selectively expressed in cartilage. Inactivation of the Nkx3.1 gene produced no apparent skeletal phenotype, while knockout of its paralogue Nkx3.2 in mice led to a lethal skeletal dysplasia, with abnormalities of the vertebral column and craniofacial bones [77–80]. *Nkx3.1* and *Nkx3.2* double knockout mice showed enhanced vertebral defects compared with *Nkx3.2* knockout mice, suggesting that *Nkx3.1* and *Nkx3.2* regulate skeletal development coordinately [81]. In addition, although knockout of some CS genes did not produce skeletal defects in mice, such mutations in humans produced skeletal disorders. As an example, knockout of *Wisp3* in mice did not yield skeletal abnormalities, while human loss-of-function mutations in *WISP3* produce the autosomal recessive skeletal disease progressive pseudorheumatoid dysplasia in humans (OMIM 208230). Depletion of the gene in zebrafish affected mandibular and pharyngeal cartilage development and over-expression *Wisp3* in zebrafish indicated that it might function as a BMP and Wnt signaling modulator [82, 83]. As another example, *COMP* encodes a noncollagenous extracellular matrix protein that interacts with cartilage collagens and promotes formation of well-defined collagen fibrils [84]. In contrast to *COMP* knockout mice, which showed normal skeletal development, dominant missense mutations of human *COMP* result in autosomal dominant pseudoachondroplasia and multiple epiphyseal dysplasia type 1 [29, 85, 86]. Furthermore, pseudoachondroplasia knock-in mice carrying the *COMP* p.Thr583Met mutation that causes a skeletal phenotype in humans exhibited short-limb dwarfism and severe degeneration of articular cartilage [87]. COMP p.Asp469del knock-in mice developed a progressive short-limb dwarfism and hip dysplasia, with disorganized growth plate [88]. Finally, dominant structural mutations in *TRPV4*, which encodes a Ca^2+^ permeable ion channel that responds to chemical and physical stimuli, result in a broad spectrum of inherited skeletal disorders in humans [14, 28, 89–91]. *Trpv4* knockout mice showed bladder dysfunction and hearing loss without obvious skeletal defects [92]. However, *Trpv4* transgenic mice expressing the known pathogenic missense mutation p.Arg594His showed a severe skeletal dysplasia that recapitulated abnormalities of metatropic dysplasia in humans [93]. In the latter examples, dominant negative or gain-of-function mutations interrupt normal chondrocyte activities and lead to abnormal skeletal phenotypes that mouse knockouts did not reveal.

### Limitations and future directions

The goal of this study was to identify key genes and transcriptional regulatory mechanisms in chondrogenesis using total RNA isolated from fetal distal femur cartilage, including all types of chondrocytes from the growth plate. As a consequence, we were not able to determine if the selective genes are uniformly expressed throughout the growth plate or are preferentially expressed in certain chondrocyte cell type(s). For example, it is known that *SOX9* is expressed in reserve and proliferating chondrocytes and *COL10A1* is expressed in hypertrophic chondrocytes [94, 95]. Laser capture microdissection followed by RNA-seq could further define the expression patterns of the cartilage-selective genes. In addition, due the limited number of tissue types and samples used in this study, and objective but arbitrary criteria for identifying cartilage selectivity, the CS gene list could change if the criteria for identifying CS selective genes are changed, or as additional fetal tissue expression profiles are added. Curation of additional RNA-seq data from human tissues and developmental stages will help to better define tissue selective genes and promote functional studies at spatial and temporal scales.

## Conclusions

In summary, the current study identified 332 protein coding genes and 34 lncRNA genes that are selectively expressed in human fetal cartilage. Proteins transcribed from the cartilage-selective genes formed a complex interaction network and participate in multiple processes in skeletal development. The data further indicate that transcription factors, enhancers and lncRNAs work cooperatively to mediate transcriptional regulation in cartilage. In addition to known regulatory molecules, our study also discovered novel protein coding genes and lncRNAs that are likely to regulate chondrogenesis. Together, these findings help us to better understand the mechanisms underlying chondrogenesis and will be instrumental in further dissecting gene regulatory mechanisms in skeletal pattern formation, endochondral ossification and chondrocyte function.

## Methods

### Cartilage specimen collection and RNA isolation

Under a UCLA IRB-approved human subjects protocol, five independent 14–18 week distal human femur growth plate cartilage samples were obtained with informed consent. RNA was isolated and purified from the tissue as previously described [96]. Briefly, the fetal cartilage was digested in 0.3% bacterial collagenase, the chondrocytes were collected by centrifugation and total RNA was isolated with TRIZol (Thermo Fisher Scientific). Genomic DNA contamination was removed by DNase I (Thermo Fisher Scientific) digestion and the RNA was further purified with the RNeasy Mini Kit (Qiagen). All of the RNA samples had RIN (RNA Integrity Number) ≥8 (measured with the Agilent 2100 BioAnalyzer).

### Gene expression analysis

PolyA-tailed mRNA was isolated from total RNA and used as the input for sequencing library construction with the TruSeq RNA Preparation Kit (Illumina). Sequencing was performed on the Illumina HiSeq 2000 instrument (Additional file 1: Dataset S1). The sequencing data analysis pipeline was described previously [97]. Briefly, RNA-seq reads were mapped to the human genome (hg19) using TopHat (v2.0.9), allowing up to 2-bp mismatches per read [98]. The mapped reads were analyzed using the Cufflinks (v2.2.1) package [99]. The transcript abundance in each sample was quantified with Cuffquant. The Cuffquant outputs for all samples were then joined and normalized with Cuffnorm. Gene expression level is represented as FPKM (fragments per kilobase of exon per million fragments mapped) values. Refseq gene annotation was obtained from the UCSC genome website (https://genome.ucsc.edu/). LncRNA gene annotation was downloaded from GENCODE [100]. Low abundance genes with FPKM values less than 1 were filtered before downstream analysis.

### Identification of cartilage-selective (CS) genes

The PaGeFinder program has been used to define gene expression patterns from high-throughput transcriptomic data, which applies cosine similarity measurements to discover housekeeping and tissue-specific/selective genes [101]. The specificity measure (SPM) value produced by PaGeFinder was used as a criterion to estimate the relative expression specificity of a gene in a given sample. To identify CS genes, gene expression FPKM values from multiple samples within the same tissue/cell type were averaged, yielding a table with average FPKM values for all measured genes. The data were then processed with PaGeFinder and SPM values were generated. CS genes were defined as the subset of genes with average FPKM values ≥1 and SPM ≥0.9 in cartilage samples. For the cartilage-selective lncRNAs, to eliminate false-positives, lncRNAs that had sense strand exonic overlap with expressed protein-coding genes or that were embedded in gene introns for which there was intron-retention, were excluded from the cartilage-selective lncRNA list.

### External data sources

The data used in this study are summarized in Additional file 1: Dataset S1. Some of the RNA-seq data from normal human tissues/cells have been reported by others [17–23]. Histone modification ChIP-seq data were downloaded from the NCBI epigenome roadmap project (http://www.ncbi.nlm.nih.gov/geo/roadmap/epigenomics/) [20]. Processed alignment files for histone ChIP-seq in human fetal tissues were also downloaded from the epigenome roadmap project website (http://egg2.wustl.edu/roadmap/data/byFileType/alignments/consolidated/). Genome-wide SOX9 binding data in mouse chondrocytes were described previously [52]. All downloaded data were processed using the UCSC hg19 build.

### ChIP-seq data processing

Bowtie 0.12.7 was used to align sequenced reads to the human (hg19, for histone modifications) and murine genomes (mm9, for SOX9 ChIP-seq). Reads that aligned to more than one genomic location or that had more than 2 mismatches were discarded. The human genome was divided into 100 bp windows and peak calling was performed with MACS (v1.3.7.1) using its default *P*-value [102].

### Prediction of active enhancers

Active enhancers were predicted based on H3K27ac ChIP-seq peaks that were >3 kb away from gene transcription start sites (TSS) and H3K4me3 peaks (a marker for active promoters) [55]. Enhancers within 1 kb of each other were merged. Chondrocyte-unique enhancers were defined as the enhancers only detected in chondrocytes in comparison with other tissues.

### Gene ontology (GO) and network analysis

CS genes were submitted to DAVID for GO and KEGG pathway analysis [103]. GO analysis for chondrocyte-unique enhancers was performed with the Genomic Regions Enrichment of Annotations Tool (GREAT) using default settings [56]. Genome coordinates for CS lncRNAs were submitted to GREAT and protein coding genes flanking lncRNA loci were identified. Gene network analysis was performed with Cytoscape 3.2.1, using plug-ins ClueGO and GeneMANIA [31, 104, 105].

### Motif analysis

Enrichment analysis of known transcription factor binding motifs was performed with SeqPos [106] using DNA sequences from gene promoter regions (±2 kb from TSS) or enhancers.

### Data processing and visualization

Heatmaps, boxplots and scatter plots were generated with R. Sequencing data were visualized with the Integrated Genome Browser [107].

## Additional files

Additional file 1: Supplementary Dataset S1. Data used in the study. (XLSX 17 kb)

Additional file 2: Figure S1. Dendrogram and heatmap of the Pearson’s correlation coefficient for human tissue gene expression (Log_2_FPKM). Only genes with FPKM > 1 in at least one sample were used for analysis. The cartilage samples are highlighted with a red frame. **Figure S2.** Box plots showing cartilage-selective gene expression in cartilage (red) and non-cartilage (blue) tissues. The thick horizontal line in each box represents the median gene expression value. **Figure S3.** Bar graph showing *RUNX2* expression in human tissues. Red bars indicate cartilage samples and blue bars indicate non-cartilage samples, respectively. Gene expression levels are shown as FPKM values. **Figure S4.** Genome browser views of H3K4me1 and H3K27ac peaks along **a** *COL2A1* and **b** *COL11A1* loci. Chromosome coordinates are shown as black bars on top. Black arrows indicate the direction of transcription on a diagram of each gene below. CH, chondrocytes. AG, adrenal gland. LI, large intestine. SI, small intestine. MU, muscle. SC, spinal cord. ST, stomach. TH, thymus. **Figure S5.** Genome browser views of H3K4me1, H3K4me3, H3K27ac peaks and RNA expression track along lncRNA loci (A) *AC058791.1* and (B) *RP11–261P24.2* in human chondrocytes. **Figure S6.** Sox9 binds to the lncRNA *2610035D17Rik* promoter and gene body in mouse rib chondrocytes. **a** Genome browser view of Sox9 binding along *Sox9* and *2610035D17Rik* loci on chromosome 11. **b** Shaded region in **a** was enlarged for a close view of Sox9 binding at the *2610035D17Rik* promoter and first intron. (PDF 912 kb)

Additional file 3: Supplementary Dataset S2. Cartilage-selective genes. (XLSX 45110 kb)

Additional file 4: Supplementary Dataset S3. Data comparison between microarray and RNA-seq platforms. (XLSX 8 kb)

Additional file 5: Supplementary Dataset S4. Cartilage-selective transcription factors and motif analysis. (XLSX 26 kb)

Additional file 6: Supplementary Dataset S5. GO analysis of cartilage-unique enhancers. (XLSX 1028 kb)

Additional file 7: Supplementary Dataset S6. Cartilage selective lncRNAs. (XLSX 17845 kb)
