# Supplementary material for: Genes uniquely expressed in human growth plate chondrocytes uncover a distinct regulatory network
Source: BMC Genomics. 2017 Dec 20;18:983. doi: 10.1186/s12864-017-4378-y (PMC5738906; doi:10.1186/s12864-017-4378-y)
Supplement: Supplementary file 2 — Dendrogram and heatmap of the Pearson’s correlation coefficient for human tissue gene expression (Log2FPKM). Only genes with FPKM > 1 in at least one sample were used for analysis. The cartilage samples are highlighted with a red frame. Figure S2. Box plots showing cartilage-selective gene expression in cartilage (red) and non-cartilage (blue) tissues. The thick horizontal line in each box represents the median gene expression value. Figure S3. Bar graph showing RUNX2 expression in human tissues. Red bars indicate cartilage samples and blue bars indicate non-cartilage samples, respectively. Gene expression levels are shown as FPKM values. Figure S4. Genome browser views of H3K4me1 and H3K27ac peaks along a COL2A1 and b COL11A1 loci. Chromosome coordinates are shown as black bars on top. Black arrows indicate the direction of transcription on a diagram of each gene below. CH, chondrocytes. AG, adrenal gland. LI, large intestine. SI, small intestine. MU, muscle. SC, spinal cord. ST, stomach. TH, thymus. Figure S5. Genome browser views of H3K4me1, H3K4me3, H3K27ac peaks and RNA expression track along lncRNA loci (A) AC058791.1 and (B) RP11–261P24.2 in human chondrocytes. Figure S6. Sox9 binds to the lncRNA 2610035D17Rik promoter and gene body in mouse rib chondrocytes. a Genome browser view of Sox9 binding along Sox9 and 2610035D17Rik loci on chromosome 11. b Shaded region in a was enlarged for a close view of Sox9 binding at the 2610035D17Rik promoter and first intron. (PDF 912 kb) [file 12864_2017_4378_MOESM2_ESM.pdf]

Figure S1

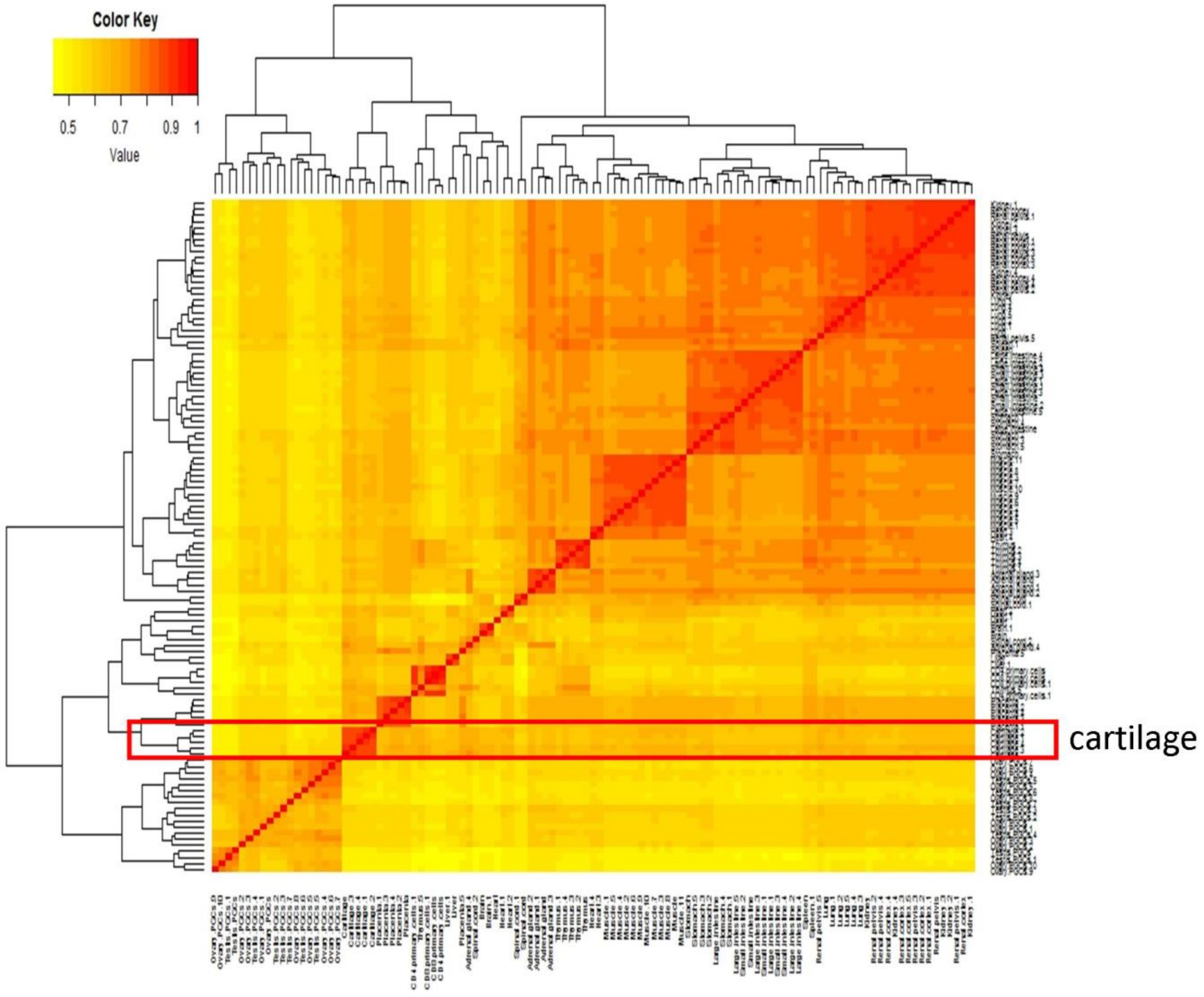

Figure S1. Dendrogram and heatmap of the Pearson's correlation coefficient for human tissue gene expression (Log_2FPKM). Only genes with FPKM>1 in at least one sample were used for analysis. The cartilage samples are highlighted with a red frame.

Figure S2

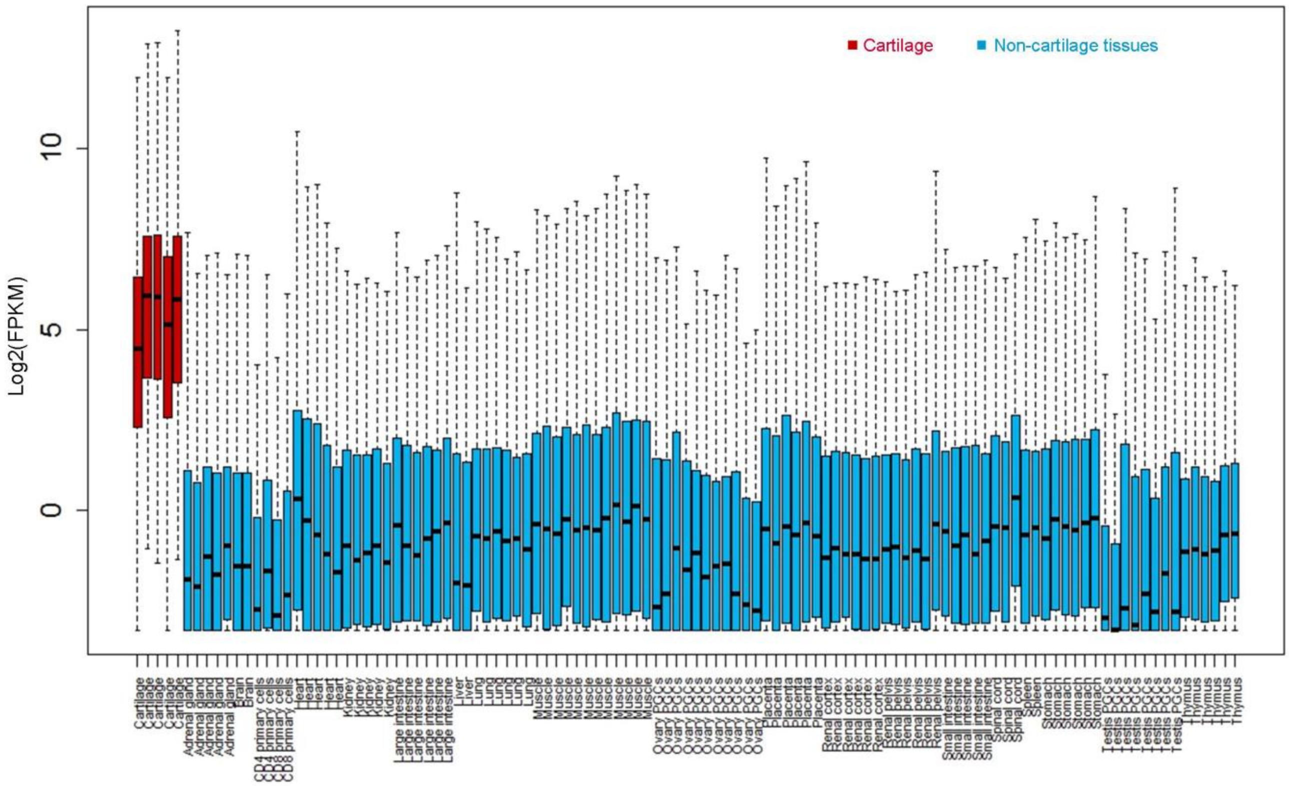

Figure S2. Box plots showing cartilage-selective gene expression in cartilage (red) and non-cartilage (blue) tissues. The thick horizontal line in each box represents the median gene expression value.

Figure S3

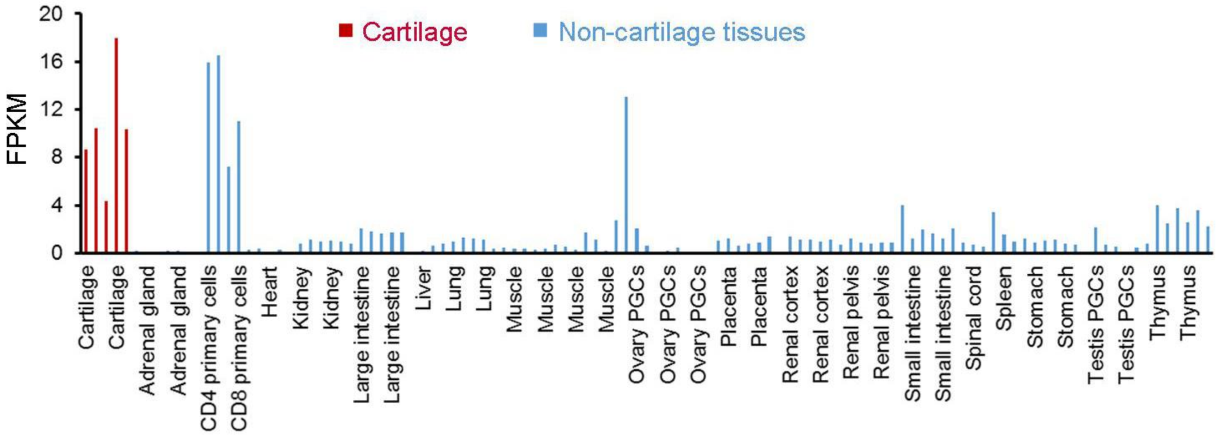

Figure S3. Bar graph showing *RUNX2* expression in human tissues. Red bars indicate cartilage samples and blue bars indicate non-cartilage samples, respectively. Gene expression levels are shown as FPKM values.

Figure S4

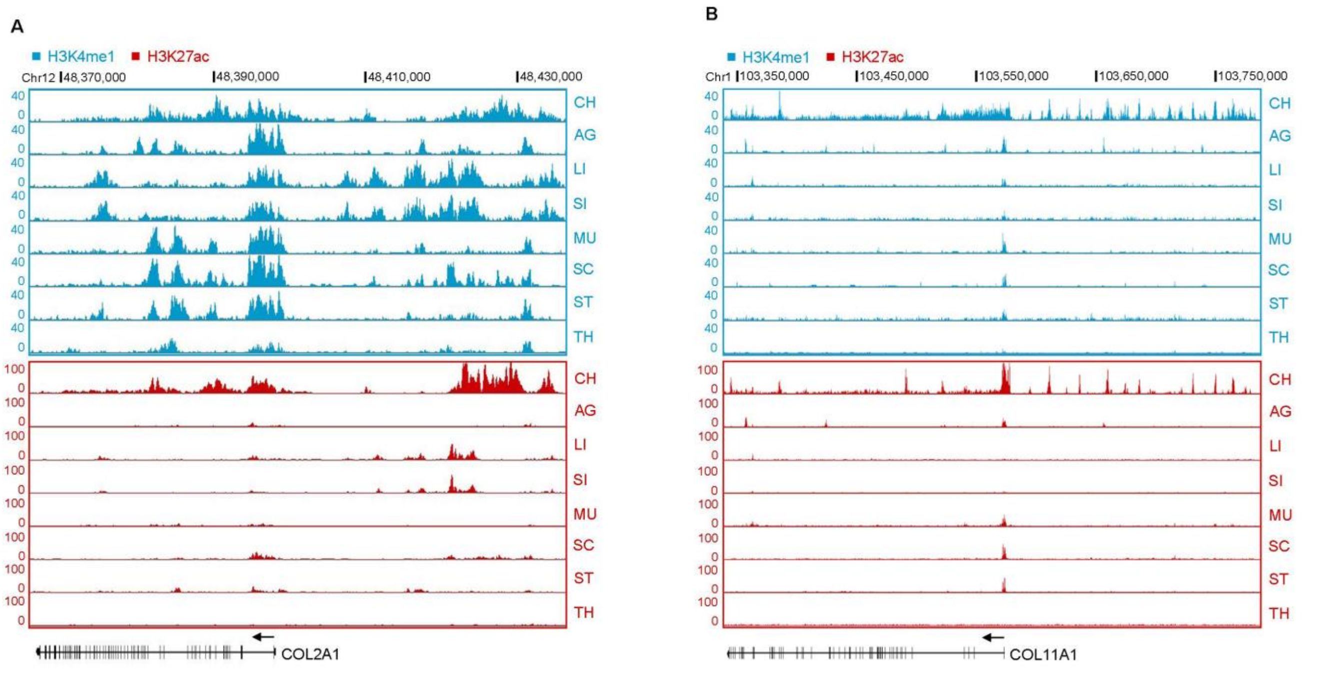

Figure S4. Genome browser views of H3K4me1 and H3K27ac peaks along **a** *COL2A1* and **b** *COL11A1* loci. Chromosome coordinates are shown as black bars on top. Black arrows indicate the direction of transcription on a diagram of each gene below. CH, chondrocytes. AG, adrenal gland. LI, large intestine. SI, small intestine. MU, muscle. SC, spinal cord. ST, stomach. TH, thymus.

Figure S5

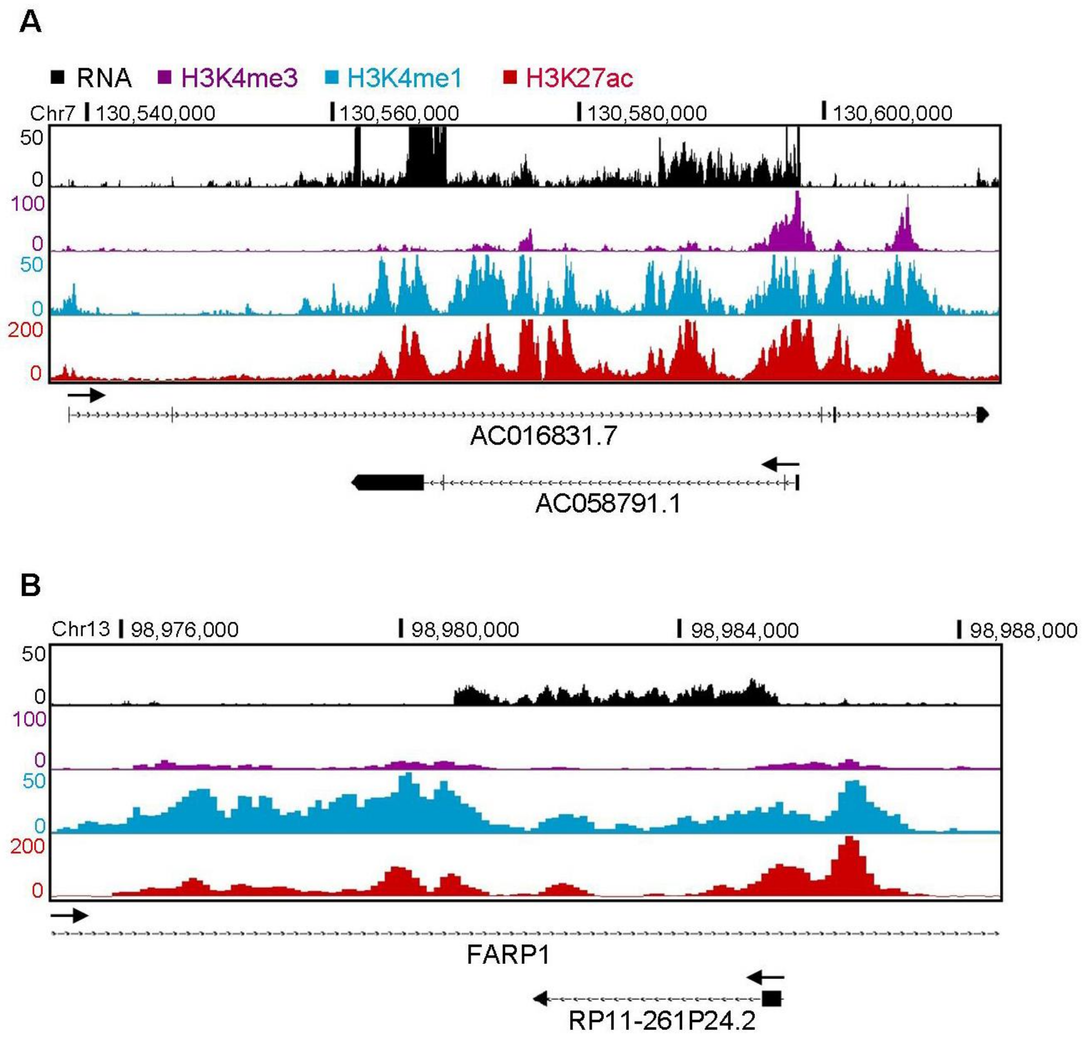

Figure S5. Genome browser views of H3K4me1, H3K4me3, H3K27ac peaks and RNA expression track along lncRNA loci (A) *AC058791.1* and (B) *RP11-261P24.2* in human chondrocytes.

Figure S6

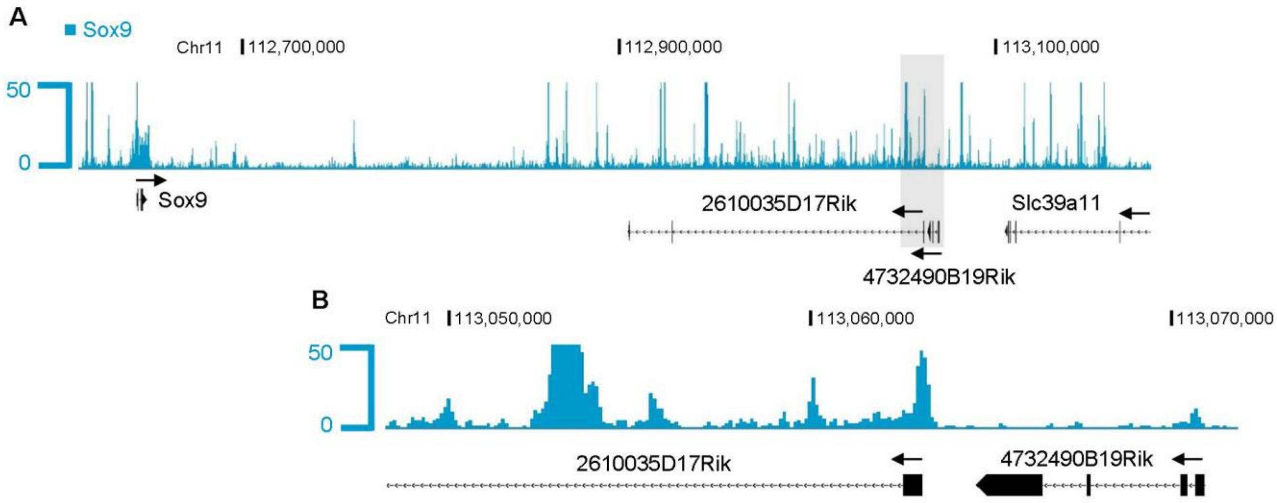

Figure S6. Sox9 binds to the lncRNA *2610035D17Rik* promoter and gene body in mouse rib chondrocytes. **a** Genome browser view of Sox9 binding along *Sox9* and *2610035D17Rik* loci on chromosome 11. **b** Shaded region in **a** was enlarged for a close view of Sox9 binding at the *2610035D17Rik* promoter and first intron.
